# Measurement of the Chair Rise Performance of Older People Based on Force Plates and IMUs

**DOI:** 10.3390/s19061370

**Published:** 2019-03-19

**Authors:** Sandra Hellmers, Sebastian Fudickar, Sandra Lau, Lena Elgert, Rebecca Diekmann, Jürgen M. Bauer, Andreas Hein

**Affiliations:** 1 Assistance Systems and Medical Device Technology, Carl von Ossietzky University Oldenburg, 26129 Oldenburg, Germany; sandra.lau@uni-oldenburg.de (S.L.); rebecca.diekmann@uni-oldenburg.de (R.D.); andreas.hein@uni-oldenburg.de (A.H.); 2 Peter L. Reichertz Institute for Medical Informatics, TU Braunschweig and Hannover Medical School, TU Braunschweig, 38106 Braunschweig, Germany; lena.elgert@plri.de; 3 Center for Geriatric Medicine, University Heidelberg, 69117 Heidelberg, Germany; juergen.bauer@bethanien-heidelberg.de

**Keywords:** chair rise test, machine learning, geriatric assessment, wearable sensors, IMU, functional decline, sit-to-stand

## Abstract

An early detection of functional decline with age is important to start interventions at an early state and to prolong the functional fitness. In order to assure such an early detection, functional assessments must be conducted on a frequent and regular basis. Since the five time chair rise test (5CRT) is a well-established test in the geriatric field, this test should be supported by technology. We introduce an approach that automatically detects the execution of the chair rise test via an inertial sensor integrated into a belt. The system’s suitability was evaluated via 20 subjects aged 72–89 years (78.2 ± 4.6 years) and was measured by a stopwatch, the inertial measurement unit (IMU), a Kinect^®^ camera and a force plate. A Multilayer Perceptrons-based classifier detects transitions in the IMU data with an F1-Score of around 94.8%. Valid executions of the 5CRT are detected based on the correct occurrence of sequential movements via a rule-based model. The results of the automatically calculated test durations are in good agreement with the stopwatch measurements (correlation coefficient r = 0.93 (*p* < 0.001)). The analysis of the duration of single test cycles indicates a beginning fatigue at the end of the test. The comparison of the movement pattern within one person shows similar movement patterns, which differ only slightly in form and duration, whereby different subjects indicate variations regarding their performance strategies.

## 1. Introduction

**Figure 1:**
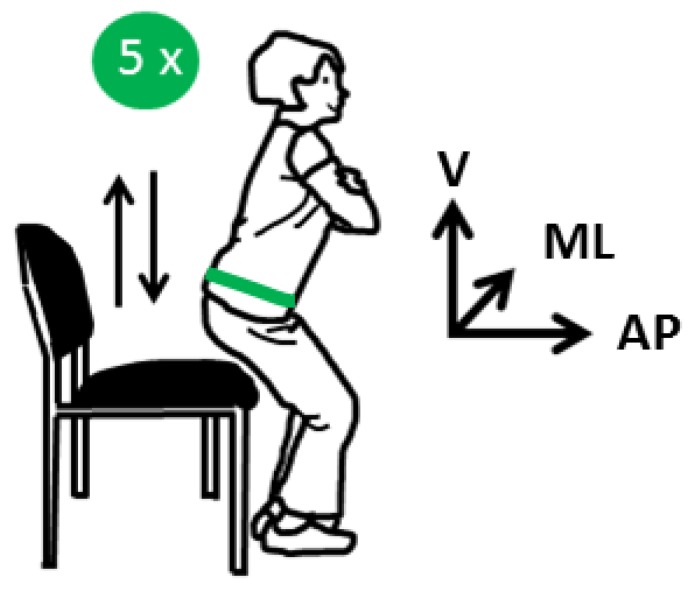
Besides a chair and a stopwatch or an inertial measurement unit (IMU), nothing else is needed for the chair rise test. The orientation of the hip-worn IMU is illustrated in this figure (vertical (V), mediolateral (ML), anterior-posterior (AP).

Facing the challenge of the demographic shift, the maintenance of the independence of older adults becomes more and more important [1]. Therefore, preventive measures of physical function are necessary to identify functional decline early and to start intervention programs (such as individual fitness trainings) and to reduce the risk of downstream decline [2]. The decline can be detected via functional assessments covering different aspects of functioning such as balance, mobility, endurance and power [3]. In order to assure such an early detection, functional tests must be conducted on a frequent and regular basis and should be supported by automated technology to keep the additional burden for medical experts as low as possible. Therefore, we introduce an automated measuring system for detecting age-related functional decline. Bean et al. emphasized muscle power as an important determinant of mobility functioning in older adults [4], which can be estimated based on different tests. Among others, the chair rise test is a well-established geriatric test to assess the leg power [5] and thus to detect early age-related changes in the functional status. For the chair rise test, participants sit centered on a chair, placing their hands on the opposite shoulder crossed at the wrists (Figure 1). After the start signal, they rise to a full standing position and then sit back down again as fast as possible. This sequence is repeated several times regarding the specific test procedures (5 times, 10 times, 30 s).

The advantage of the chair rise test in comparison to other geriatric tests is its simplicity and the possibility to perform this test in clinical as well as in home environments. Even with an already existing functional impairment, patients are able to perform this test. Therefore, this test is a suitable test for our measurement system.

As another benefit, the chair rise test requires not only lower limb strength and power but also good balance and coordination [5] and, therefore, covers several components of physical function. In the traditional approach, the duration of the chair rise test is measured by stopwatches as single indicator [5,6,7]. This does not fully exploit the potential of the test as more performance parameters can be derived, which may be more medically relevant than the total test duration. However, technology is needed, in order to measure these comprehensive parameters. As a result, the interest in assessments supported by technology has risen significantly to ensure objective and more detailed analyses. A growing body of literature has examined the use of inertial measurement units (IMU) for sit-to-stand analyses [8,9,10,11,12]. However, video-based systems [13] or force plates [14,15,16] have also been utilized for analyses of chair rise tests. Additionally, various approaches have been proposed to investigate the sit-to-stand (STS) performance, especially in older people. From this, for example, the mean power can be estimated based on the test duration [6,7]. Several groups focused also on the trunk movement [8,11,16,17]. However, research has tended to focus on the sit-to-stand transition rather than the stand-to-sit transition [18], which might also hold crucial information of the functional status.

In order to introduce a novel approach of an automatic and more generalized chair rise test evaluation system for the early detection of functional decline, we present an IMU-based system that automatically detects the execution of the chair rise test via an inertial sensor integrated into a belt and analyzes this test regarding various aspects, which are mentioned in the following. In contrast to existing studies, we do not only focus on the evaluation of the sit-to-stand transition parameters but also consider differences during the single cycles of chair rise test and therefore reveal the characteristic fatigue and unsteadiness associated with the test, which is not considered in most studies. In addition, in this article, different performance strategies of older adults within the five time chair rise test (5CRT) are considered, which enables a more generalized investigation of the chair rise performance. We compare the repeated movements by a single person as well as across different persons. These movements are monitored by a multi-sensor based assessment system, which includes inertial, optical and mechanical sensors. Our main system consists of an IMU integrated into a belt and automatically detects the single transition as well as the whole 5CRT-sequence with a machine learning classifier. After the recognition of the corresponding sequence, the system calculates different performance parameters such as the duration of each transition or of the whole test. The consideration of individual transitions is of special interest, since enabling a separate consideration for clinical assessment as potential predictors of functional decline. Since, in conventional settings, the test duration is the only parameter used to assess the performance, we thereby investigate the association of these separate transitions towards other classical geriatric assessments. Furthermore, we use a force plate and a Kinect camera as reference systems. In order to understand the performance strategies, an understanding of biomechanics of the sit-to-stand and stand-to-sit transitions is necessary. Therefore, we also focus on the biomechanical signal interpretation until we move on to performance evaluations.

## 2. Materials and Methods

In this study, the 5CRT was measured in a conventional manner with manual stopwatch measurements, and technically via a force plate, a Microsoft Kinect depth camera (Redmond, WA, USA) and a wearable sensor system. The wearable system consists of one IMU integrated into a belt. The positioning in a belt assures an unobtrusive sensor-placement and can also be easily handled by older adults at home independently.

In the 5TCRT, the participant sits on a height-adjustable chair without armrests. The legs are positioned in a 90${}^{\circ }$ angle, and the feet stand firmly on a force plate. In a conventional assessment, the time is measured to complete five cycles of rising from a chair until standing fully erect to a straight and upright position and then sitting down again with arms folded across the chest. A lower test duration means a better performance. Therefore, the corresponding cut-off values for the performance evaluation are listed in Table 1.

The 5CRT is a part of the Short Physical Performance Battery (SPPB) [20], which consists of two other tests besides the 5CRT, namely a 4 m gait speed test and balance tests (side-by-side stand, semi-tandem stand, and tandem stand). Additionally to the SPPB, several other geriatric tests (Frailty Criteria according to Fried [21], Timed up and Go Test (TUG) [22,23], 6 Minute Walk Test (6 min WT) [24], De Morton Mobility Index (DEMMI) [25,26], Stair Climb Power Test (SCPT) [27], and Counter Movement Jump [28]) were conducted in this study. The description of the complete study can be found in [29]. All tests were measured conventionally by medical professionals and additionally by our multi-sensor system. The study has been approved by the appropriate ethics committee (ethical vote: Hanover Medical School No. 6948) and conducted in accordance with the Declaration of Helsinki.

### 2.1. Measurement Systems

#### 2.1.1. Sensor Belt

**Figure 2:**
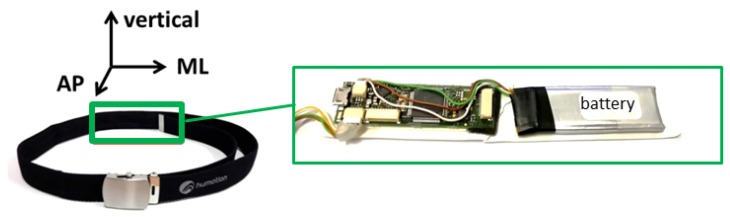
Orientation of the sensor system, which is integrated into a belt. The abbreviations stand for mediolateral (ML) and anterior-posterior (AP).

Our main system consists of a commercially available sensor-unit (DX-3, Humotion GmbH, Münster, Germany), which includes a triaxial accelerometer, gyroscope, and magnetometer, as well as a barometer. The sensor-unit is integrated into a hip-worn belt and the sampling rate is 100 Hz for all sensors. The hip positioning was chosen to enable an easy use and to assure a fixed positioning and an equal orientation of the sensor-unit among all participants. The orientation of the sensor belt is illustrated in Figure 2. Due to its light (overall weight: 140 g) and compact dimensions (11 cm × 2.5 cm including battery), the belt enables flexible, easy, and unobtrusive measurements. The correct placement of the sensor belt, as well as the position of the sensor unit inside the belt were checked and adjusted according to the hip circumference for each participant of our study by our medical professionals. The correct alignment between the L3 and L5 lumbar vertebral body was important, especially regarding our machine learning approach and a good classification performance.

#### 2.1.2. Force Plate

**Figure 3:**
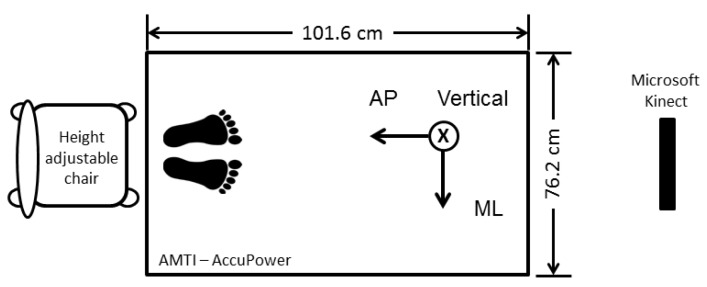
Orientation and dimension of the AMTI AccuPower force plate. The chair for the five time chair rise test is placed beside the plate. The abbreviations stand for mediolateral (ML) and anterior-posterior (AP). A Kinect camera is used as optical reference system.

Force plates measure the ground reaction force (GRF), which acts on the plate. Figure 3 shows the orientation and dimensions of the AccuPower force platform (Advanced Mechanical Technology, Inc. (AMTI), Watertown, MA, USA) used in our study. The main force acts perpendicular to the plate in the vertical direction. However, forces in the mediolateral direction and in the anterior–posterior direction are also taken into account because they might hold noteworthy information about the balance ability. The force plate in our study measures with a sample rate of 200 Hz. The subject sits on a height-adjustable seat beside the force-plate while his feet stand firmly on the plate.

#### 2.1.3. Microsoft Kinect

**Figure 4:**
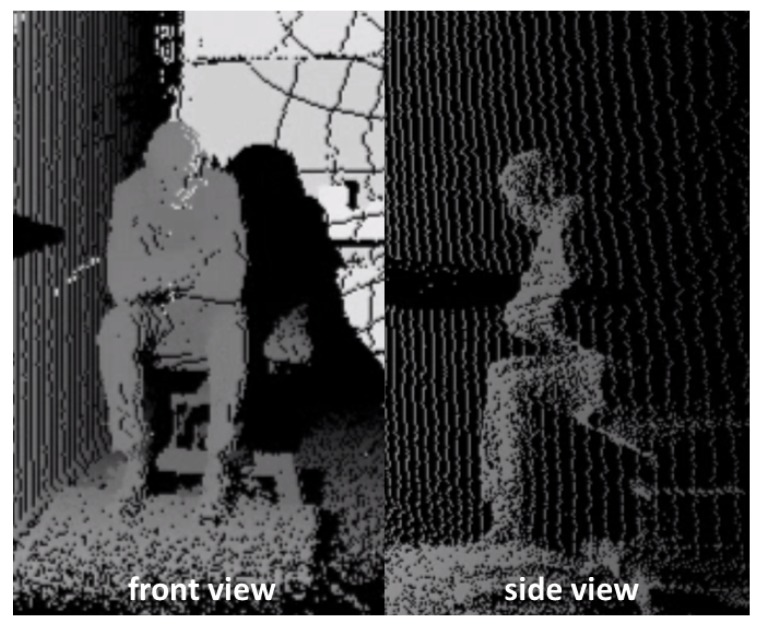
Kinect depth images of the chair rise test in front and side views.

A Microsoft Kinect V2 depth camera (30 fps) was positioned in front of the force plate and the chair. The Kinect depth data is used as optical reference system, especially for the biomechanical signal interpretation. Figure 4 shows an example of the depth images in front view and a calculated side view.

To ensure a precise synchronization of the data time across all used devices, every computer synchronizes its system time via the Network Time Protocol (NTP) with an NTP server. However, especially, for the direct comparison of the measurements of different system, the synchronization was also checked by significant points in the signals.

### 2.2. Machine Learning Classification-Model for Activity

**Figure 5:**
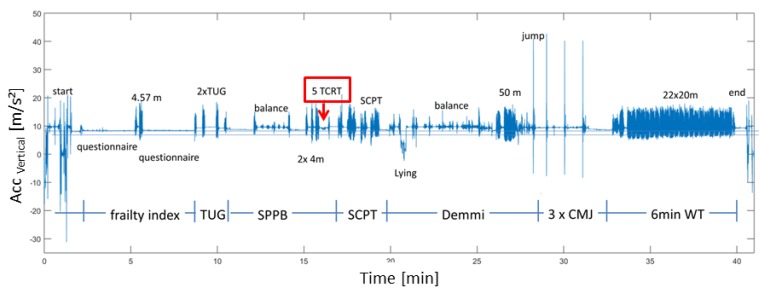
Acceleration in vertical direction during the functional assessments in our study: Frailty Criteria, Timed Up and Go (TUG), Short Physical Performance Battery (SPPB) including five times Chair-Rise-Test (5CRT), Stair Climb Power Test (SCPT), de Morton Mobility Index (DEMMI), the counter movement jump (CMJ) and the 6 min walk test (6 min WT).

Figure 5 shows the acceleration in the vertical direction during the functional assessments in our study. The 5CRT is marked in red. In order to realize an automated 5CRT analysis based on the IMU data, we use a hierarchical machine learning (ML) algorithm. First, the state of the activity (static, dynamic, transition) is classified. The subsequent classifier recognizes the relevant movements (namely, sitting (static), standing-up (transition), standing (static) and sitting down (transition)). We therefore trained classification models under consideration of various ML-approaches (Boosted Decision Trees (BDT), Boosted Decision Stumps (BDS), Multilayer Perceptrons (MLP), Adaptive Multi-Hyperlane Machine (AMM)), features and sliding window parameters. A five-fold cross validation was used to validate the performance of our classifier. The complete study and more details of our machine learning algorithm can be found in [30].

We used the following features in our study:

- Root Mean Square (RMS),
- Mean,
- Signal Energy (SE),
- Auto Correlation (AC),
- Correlation (C),
- Signal Magnitude Area (SMA),
- Standard Deviation (SD),
- Pitch.

**Figure 6:**
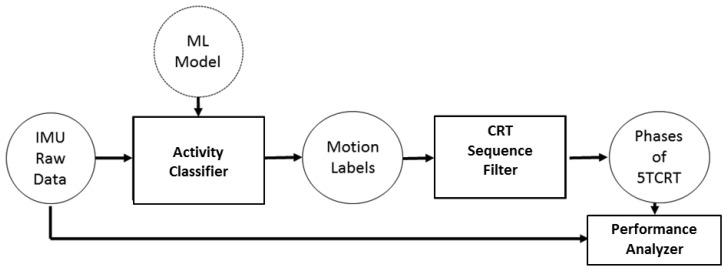
Classification- and 5CRT detection-workflow. The system combines a machine-learning based activity classifier, a rule-based model for test detection and algorithms for performance analyses.

A detailed description of each feature and its calculation can be found in [31]. The different approaches were evaluated by the F1-Score, which was calculated by following equations:

(1) $$F1=2\cdot \frac{precision\cdot recall}{precision+recall},$$

where precision and recall are defined by:

(2) $$precision=\frac{tp}{tp+fp}$$

and

(3) $$recall=\frac{tp}{tp+fn},$$

with *tp* as true positives, *fp* as false positives and *fn* as false negatives. Additionally, a low-pass filter (cut-off frequency: $f_{c}=4.5$ Hz) was used for noise reduction. Further details are discussed in [30]. Figure 6 shows the resulting classification- and 5CRT detection-workflow.

In order to automatically detect sequences of 5CRT sequences, the annotations resulting from the ML classifier are processed regarding the occurrence of sequential activities via the following rule-based approach. Therefore, we check the motion labels, which were automatically created by our machine learning algorithm and search for valid test sequences. A valid sequence consists of five iterations of the SIT_STAND label directly followed by STAND_SIT label:

5x:SIT_STAND→STAND_SIT.

Since the participants are allowed to hold the position of sitting or standing for a while during the test, the labels SIT and STAND between the corresponding transitions are also valid:

5x:SIT_STAND→STAND→STAND_SIT→SIT.

All other motion labels are not allowed during the test interval. After one valid sequence is found, the analysis algorithm will be executed for a performance assessment.

### 2.3. Statistical Data Analysis

The data of the automatically recognized CRT-sequence is analyzed in various ways. A linear regression, as well as a Bland–Altman plot, is used to analyze relationship between the overall test duration measured by the IMU-based system and by a stopwatch.

For estimating relationships among different CRT-parameters (duration of 1st/last/average sit-to-stand, 1st/last/average stand to sit, and whole test duration measured by the IMU-system) and other geriatric tests (duration of Timed up and Go (TUG), walk test of the Frailty Criteria and the Stair Climb Power Test (SCPT) measured by a stopwatch), a linear regression analysis is utilized and the significance evaluated by the *p*-value, whereby a *p*-value < 0.005 indicates a significant relation. A normalized cross-correlation measures the similarity of two signals and is used to examine the intra- and interpersonal variability in performing the CRT-cycles. The results were evaluated regarding the Rule of Thumb for interpreting the size of a correlation coefficient [32]: 1–0.9 very high correlation, 0.7–0.9 high correlation, 0.5–0.7 moderate correlation, 0.3–0.5 low correlation, and 0–0.3 negligible correlation.

## 3. Results

At the beginning of this section, we present the study population and the results of our machine learning algorithm to detect the five time chair rise test in our raw data. In order to understand the biomechanics of the detected chair rise test cycles, we first focus on the signal pattern and the qualitative progress of the movements until we move on to performance analysis. After the calculation of the temporal test parameters and their evaluations, a comparison of the performance strategies within our study population follows.

### 3.1. Study Population

Overall, 20 healthy participants aged 72–89 years (78.2 ± 4.6 years) performed the 5CRT. The characteristics of the study population are listed in Table 2.

### 3.2. Sensitivity and Specificity of Applied Machine Learning Classifiers

Among the considered machine learning approaches (Boosted Decision Trees (BDT), Boosted Decision Stumps (BDS), Multilayer Perceptrons (MLP), Adaptive Multi-Hyperlane Machine (AMM)), the best results for the recognition of the state were achieved with a Boosted Decision Tree and for static activities with a Multilayer Perceptrons approach. Details of the classifier can be found in Table 3.

The best results for transitions (sit-to-stand, stand-to-sit) were achieved with a Multilayer Perceptrons classifier (four hidden layers, 40 hidden nodes) and a window size of 1.135 s and a step width of 0.073 s. The obtained F1-Score for state-classifier is 96.6, for the static-classifier 97.3 and for the transition-classifier is 94.8.

### 3.3. Evaluation of Overall 5CRT Duration Accuracy

The calculated 5CRT duration from the IMU-based classification results have been compared with the corresponding manual stopwatch measures for the considered 20 participants. As we have shown in [29], the stopwatch measures have a low inter-rater variability and thus represent a suitable reference measure. Regarding the classification results, the 5CRT duration was calculated as the sum of the durations for the considered sub-activities (sit-to-stand, standing, stand-to-sit and sitting) as described in Section 2.2.

**Figure 7:**
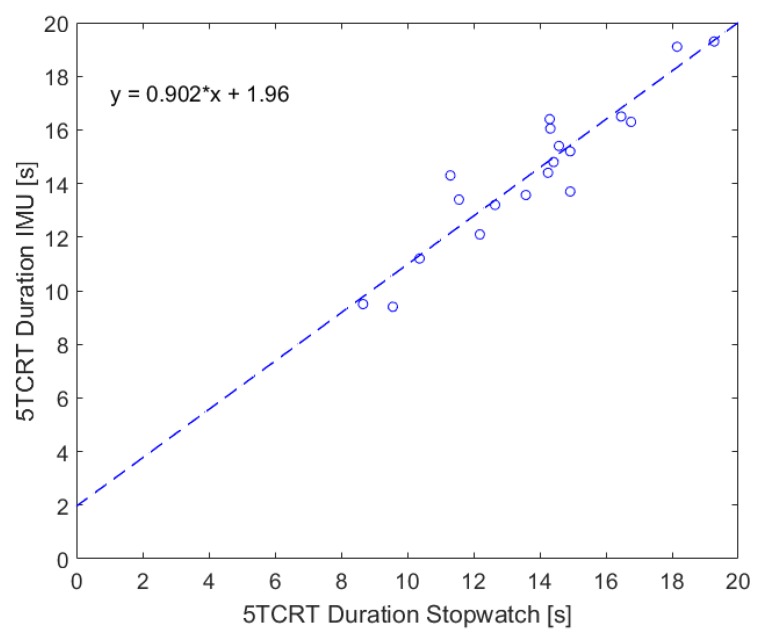
Comparison between stopwatch and IMU measurements.

The results of a linear regression among the IMU and stopwatch measurement are shown in Figure 7 and confirm a strong association—resulting in following equation:

$$t_{IMU}=0.902\cdot t_{stopwatch}+1.96.$$

In addition, a very strong correlation with r = 0.93 (*p* < 0.001) has been identified via Pearson as well.

**Figure 8:**
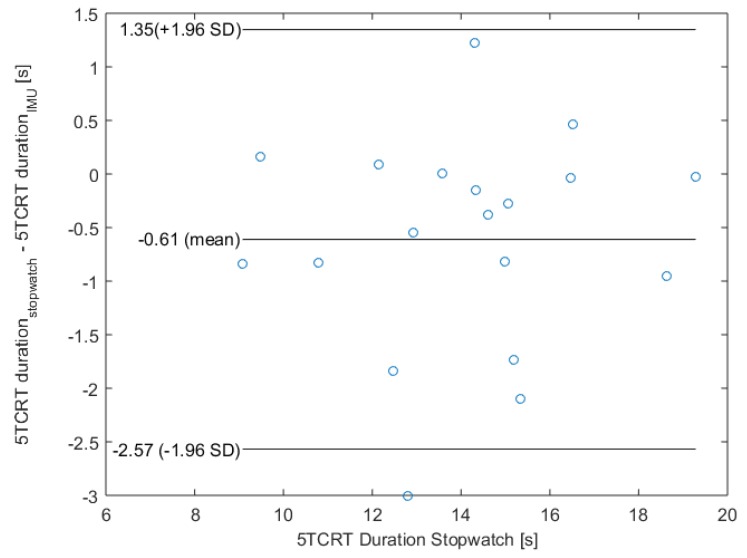
Bland–Altman plot and its characteristic values.

To evaluate the medical sensitivity, we analyzed the classification regarding the cut-off values for the 5CRT. Therefore, we considered the bias of the IMU data under using the equation of the linear regression. Only one participant was classified in the wrong category (stopwatch: 1 point, IMU: 2 points), the other 19 participants were classified correctly (2 × 1 point, 8 × 2 points, 5 × 3 points and 4 × 4 points). This classification shows also our heterogeneous study population, which includes all point categories except those who were unable to complete five cycles (0 points). Additionally, a Bland–Altman plot was used to analyze the agreement between both systems (see Figure 8). Since the mean value is −0.61, our IMU-based system has a slight fixed bias.

### 3.4. Evaluation of Distinct Transitions

**Figure 9:**
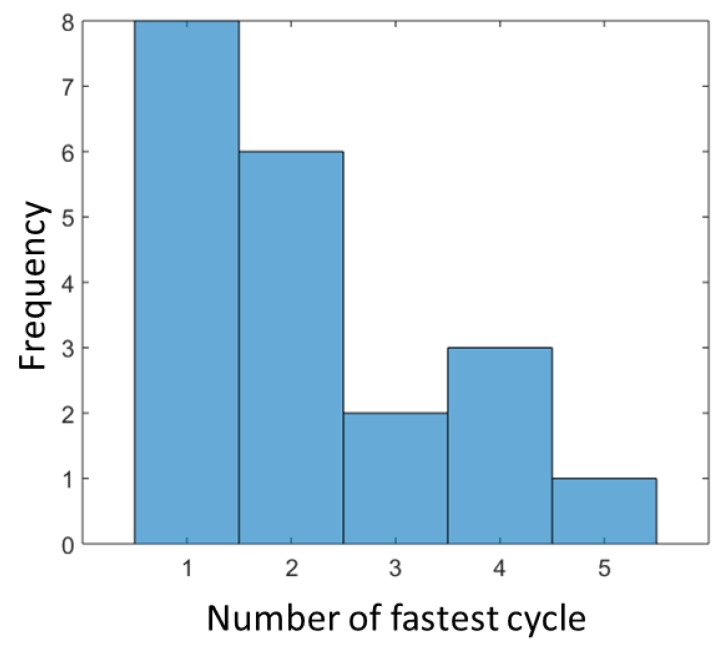
Histogram of the distribution of the fastest cycle among all five cycles.

**Figure 10:**
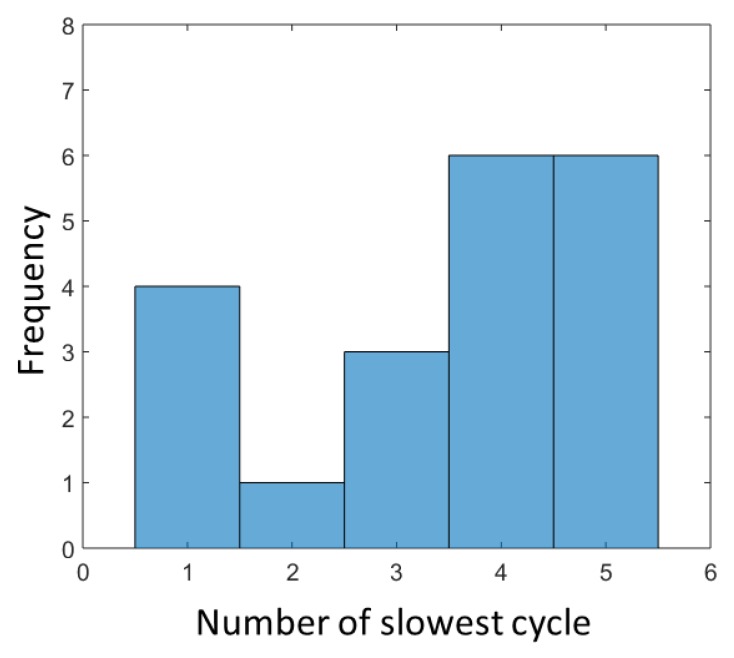
Histogram of the distribution of the slowest cycle among all five cycles.

Besides the total test duration, the duration of the single cycles and distinct transitions might be an indicator for functional decline. In order to identify differences in the performance of each cycle, we evaluated the duration of the single cycles. Figure 9 shows a histogram of the distribution of the fastest cycle among all five cycles over all subjects. In most cases, the first cycle has the shortest duration, followed by cycle two. In contrast, Figure 10 shows a histogram of the slowest cycle, which is clearly one of the last two cycles in a sequence of five cycles.

Besides the overall 5CRT duration, the evaluation of the single transitions could be worthwhile. Therefore, we calculated the duration of each transition (sit-to-stand and stand-to-sit) automatically with our machine learning classifier. A linear regression shows the relationship between our response variables and the Timed Up and Go (TUG) test duration, the Stair Climb Power Test (SCPT) and the duration of the walking test of the Frailty Criteria (distance: 4.57 m), which are other well-established tests or parts of functional geriatric tests. We analyzed the following response variables: the first sit-to-stand duration due to its standardized execution with the forced equal starting position, the last sit-to-stand duration due to the beginning fatigue, the first and last (fourth) stand-to-sit duration, the average sit-to-stand duration (over five cycles) and the average stand-to-sit duration (over four cycles because the test ends after the fifth sit-to-stand-movement) and the overall 5CRT duration. Table 4 lists the *p*-values of our regression analysis. Among these variables, only the first and average sit-to-stand duration show a significant relationship (*p*-value < 0.05) to the TUG test, with resulting regression lines of $t_{TUG}=\left(1.80\pm 0.84\right)\cdot t_{1st\;sit-to-stand}+\left(5.72\pm 1.53\right)$ and $t_{TUG}=\left(1.72\pm 0.87\right)\cdot t_{average\;sit-to-stand}+\left(5.73\pm 1.64\right)$. The stand-to-sit and the overall duration have no significant relationship to the other tests as well as the durations of the last transitions. The TUG test is the only test among the mentioned ones which includes transitions and thus, a relationship was obvious.

### 3.5. Signal-Pattern and Qualitative Progress of the Movements

**Figure 11:**
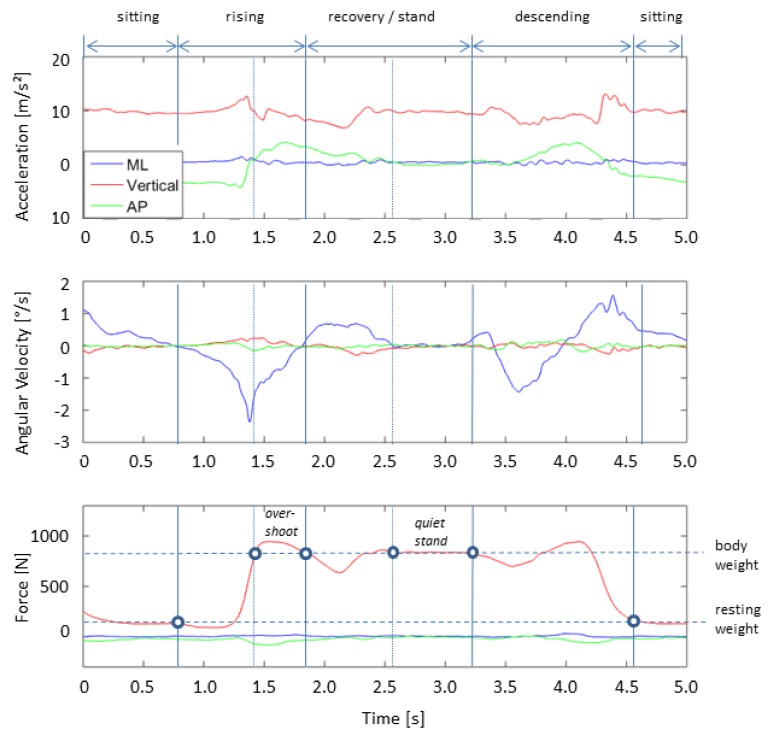
Pattern of a typical chair rise test cycle. The figure above shows the accelerometer data in the three directions over time. The figure below shows the force over time during the same sequence. The single phases are marked above.

Furthermore, the signal-patterns and the qualitative progress of the CRT movements have been studied. The pattern of a sit-to-stand and stand-to-sit cycle is shown in Figure 11 and represents the acceleration data of the IMU in mediolateral (ML), vertical and anterior-posterior direction (AP) during this sequence. Due to the acceleration of gravity, the vertical acceleration amounts to $Acc_{vertical}\approx$ 9.81 m/s${}^{2}$. The second graph shows the angular velocity of the IMU during the same sequence. The third graph shows the force during the same sequence measured by the force plate. The resting weight (weight on the plate while sitting on the chair), as well as the body weight, is marked in the figure.

The specific phases of the chair rise test cycle are marked in this figure based on the force plate data (see marker). The Kinect camera data was used as a reference system. In the first phase, the subject is sitting on a chair. The legs should form a right angle, and the feet stand firmly on the ground. During this phase, the accelerations and the forces are almost constant. The second phase describes the rising with a short phase of preparation in which the subject gains momentum and lifts his feet from the ground with a light backward movement before he shifts his weight forward in order to raise from the chair. The force increases to a maximum at this point. The rising phase (sit-to-stand) ends when the vertical force reaches the body weight again after de- and increasing after the peak force. During the standing and stabilization phase, the force oscillates around the body weight until the subject starts the stand-to-sit movement, which shows similar motion sequences to the rising phase.

The following parameters are often used for analysis: time to stand up, power, maximum ground-reaction force (GRF) or the overshoot of vertical GRF over body weight. In particular, the forces in mediolateral and anterior-posterior direction can be used for sway or balance analysis besides the oscillations in the vertical direction during the stabilization phase. This also applies to the acceleration in the corresponding directions. In comparison to the force plate measurements, the sensor belt also shows significant movements in anterior-posterior direction due to its placement at the hip, while the feet stand firmly on the ground during the whole sequence. The angular velocities can determine the orientations of the hip in detail during the movements.

**Figure 12:**
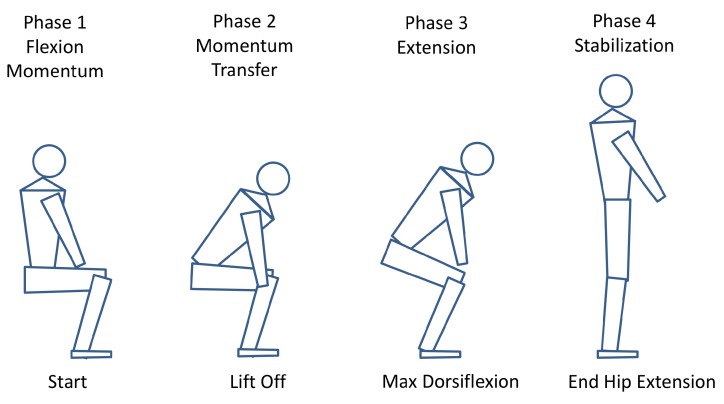
The four corresponding movement-phases of the sit-to-stand (STS) transition according to Schenkmann [35].

In a biomechanical point of view, moving from a sit-to-stand (STS) position provides complex transfer skills with adequate lower limb muscle strength and balance control. This transitional movement from sitting to upright standing posture also requires horizontal and vertical displacement of the whole body’s centre of mass (COM) from a stable to a less stable position with the COM located posterior to extended lower extremities [33,34]. During a common clinical assessment, the STS transfer can be divided into four basic phases (Figure 12). The upper body including trunk and pelvis can be described as a mobile momentum in the context of biomechanical movements. The initial Phase 1 “Flexion Momentum” starts by bending the trunk and the pelvis prior to the moment when the buttocks leave the chair base (seat-off). Continuously, in Phase 2 “Momentum Transfer”, the upper body is transferred and shifted throughout displacement of COM to the forward and upward movement of the whole body. This phase lasts from seat-off to maximum ankle dorsiflexion. An upright body position is performed during Phase 3 “Extension” and completed by the fully extended position of hip and knee joints. A straight and stable standing position characterizes the end of the transfer in Phase 4 “Stabilization” [35].

### 3.6. Investigation of Movement Patterns

Besides the duration of the whole test and the single cycles, the comparison of the movement pattern could be worthwhile. It makes sense to distinguish interpersonal and intrapersonal patterns in order to observe differences in the execution of cycles in one person and to examine differences between different persons. First, we concentrate on the differences between the different cycles of the same person.

#### 3.6.1. Intrapersonal-Variability: Cycles of the Same Participant

**Figure 13:**
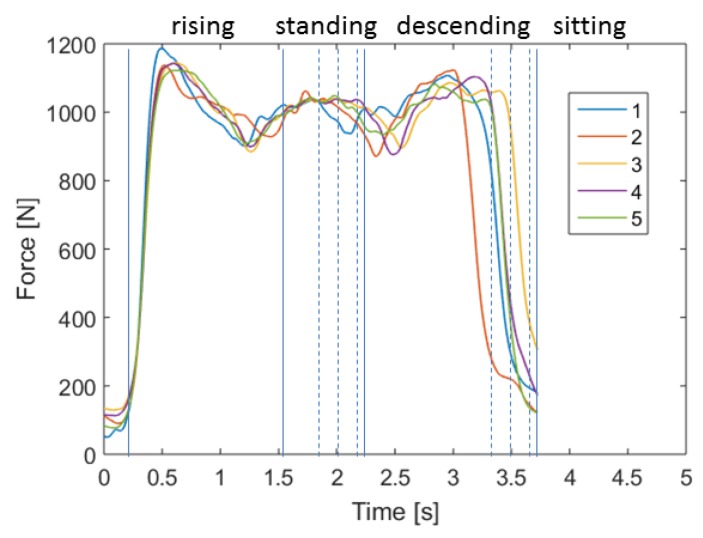
Comparison of the 5CRT cycles of one participant based on the force in vertical direction measured by the force plate.

**Figure 14:**
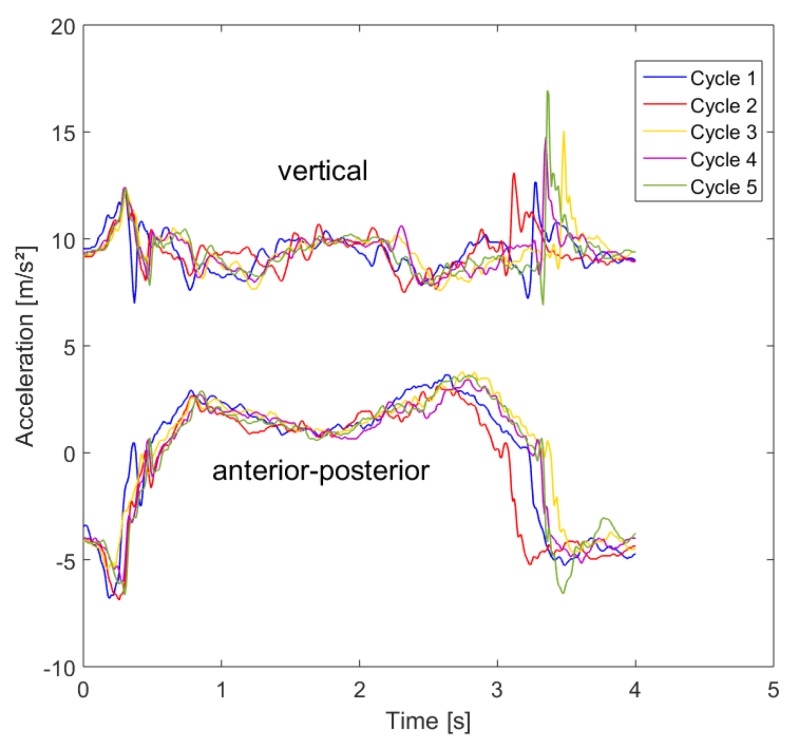
Comparison of the 5CRT cycles of one participant based on the IMU data in vertical and anterior-posterior direction.

In order to evaluate the differences in the movement pattern between the different cycles of one person, we first compared the five sit-to-stand and stand-to-sit cycles during the test. Figure 13 shows exemplarily the force plate data during the five cycles and Figure 14 the IMU acceleration data in vertical (including the acceleration of gravity) and anterior posterior direction.

For clarification purposes, the mediolateral direction (which holds the smallest changes in the amplitude) was excluded from the graph. The curves of the movements show similar patterns for both measurement systems. The variance in the duration is mostly related to the standing and descending phase and only slight differences can be seen in the form of the pattern. Therefore, the intrapersonal-variability has a low variance.

**Figure 15:**
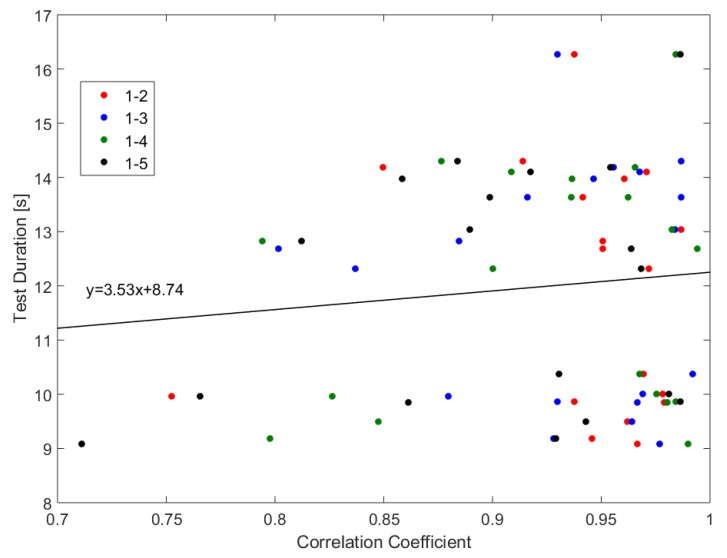
Correlation coefficient of the first test cycle with the following cycles regarding the overall test-duration.

In order to compare the pattern in a more objective point of view, we calculated the correlation coefficients by a normalized cross-correlation analysis of the first test cycle with the following cycles. The correlation coefficients on the basis of the force plate data are shown in Figure 15. In general, the evaluation shows high correlation coefficients and, therefore, a significant similarity between these patterns. This indicates that the participants use a similar performance strategy for all cycles.

Table 5 lists the statistics of the correlation coefficients of the first cycle with the subsequent cycles, which range between 0.71 and 0.99, whereby the average value is about 0.94. Regarding the Rule of Thumb for interpreting the size of a correlation coefficient [32], these results lie between a high and very high correlation. The largest deviation seems to be between the first and the last cycle with a range of 0.28. This might be an indicator of physical exhaustion. Another interesting point is that there is a slight trend for a lower similarity between the cycles of the faster participants (lower test duration) than for the slower ones, which indicates a higher intrapersonal-variability for faster performances. This can be seen by the linear regression analysis over all correlation coefficients, which results in a regression line with the equation y=3.53x+8.74.

#### 3.6.2. Interpersonal-Variability: Cycles of Different Participants

**Figure 16:**
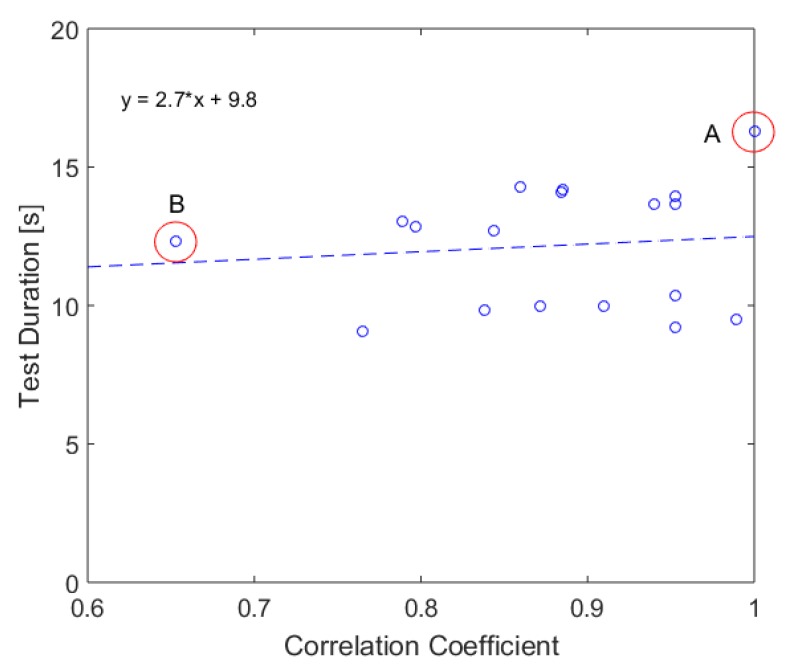
Comparison of the first 5CRT cycle of different participants based on force plate data.

Subsequently. we also tested the interpersonal-variability of the sit-to-stand and stand-to-sit pattern. We observed the first cycle due to its standardized start condition (angle of 90${}^{\circ }$). Figure 16 shows the correlation coefficients of the first cycle of participant A (slowest participant among all considered subjects) with the first cycle of the other participants. The correlation-coefficients are also determined by a normalized cross-correlation. Therefore, they describe the deviations of the form of the pattern.

Overall, this graph indicates that there exist differences in the pattern among different participants. Table 6 lists the descriptive statistics of the correlation coefficients. In comparison to the intrapersonal-variability, the correlation coefficient between different persons are significantly lower with a minimum of 0.65 and a mean correlation coefficient of 0.88.

**Figure 17:**
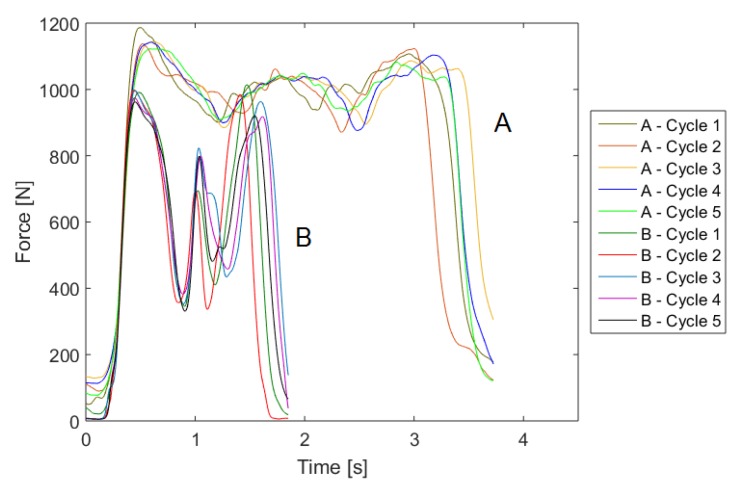
Comparison of the 5CRT cycles of participants A and B based on the force plate data.

**Figure 18:**
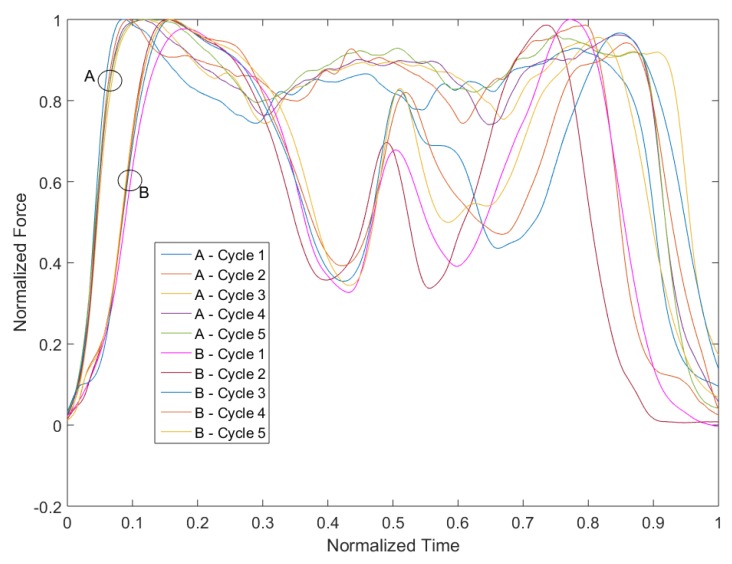
Comparison of the 5CRT cycles of participants A and B based on the force plate data with normalized axes.

In order to analyze the different strategies, we compared the pattern of participants A and B, which show the lowest correlation. Figure 17 and Figure 18 show exemplarily the force plate data (measured amplitude and time and normalized amplitude and time) during cycles one and two of participants A and B. Besides the high difference in test duration, the faster participant B shows a significantly higher dynamic in the movements (range of force). In particular, the overshoot is substantially higher than for participant A. In contrast, participant A shows an almost steady movement with a low dynamic. However, this participant has a higher swaying, which might indicate a lower balance ability. Another striking point is the higher intrapersonal-variability between the cycles of participant B, which was already mentioned in the previous subsection. A reason could be that fitter participants go more to their limits and show therefore higher physical exhaustion.

**Figure 19:**
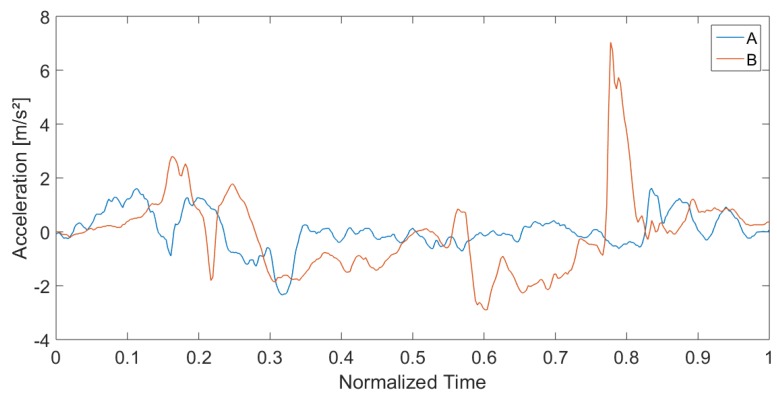
Comparison of IMU accelerometer data of participants A and B for the first cycle of the 5CRT.

Analogous to the force plate data, these findings can also be observed in the acceleration data. Figure 19 shows the normalized acceleration over all three axes for the first 5CRT cycle:

$$a_{norm}\left(t\right)=\sqrt{a_{x}{\left(t\right)}^{2}+a_{y}{\left(t\right)}^{2}+a_{z}{\left(t\right)}^{2}}.$$

The acceleration of gravity was excluded. The time axis was also normalized to focus on the differences in the form of the movements. We can observe the significantly higher dynamic in the movements of participant B and the higher swaying of participant A.

## 4. Discussion

Due to the importance of an early-stage detection of functional decline, we introduced a multi-sensor system to analyze the five time chair rise test (5CRT). This test is a well-established assessment item for the evaluation of the functional status. By an inertial-sensor based system integrated into a belt, we recognized automatically the relevant activities of the test (sit-to-stand, stand-to-sit, sitting, and standing) via a machine-learning based classifier. Valid test cycles are detected by a rule-based model. The classifier achieved good results in the detection of transitions (sit-to-stand, stand-to-sit) with an F1-Score of around 94.8%. This result is comparable with previous work. For example, Allen et al. [36] reached an accuracy of 93.1 for sit-to-stand transitions and 88.3 for stand-to-sit transition with a Gaussian mixture model and a waist-mounted triaxial accelerometer. Gupta et al. [37] achieved an accuracy of 95.4 using k-nearest neighbor (k-NN) and 97.7 using Naive Bayes for transitions. The recognition of the transitions is important for automated measurements and subsequent evaluations of the test.

Since conventional assessments usually consider the total test duration only, we investigated whether our automatic IMU-based system is also capable of calculating correct test durations and achieved good results with a significant correlation of 0.93. The medical sensitivity could also be initially confirmed since 19 of 20 participants were correctly classified regarding the cut-off values for the 5CRT. Although our heterogeneous study population included all categories (except zero points), a larger study population is needed to verify the significance of this result. Additionally, we evaluated the agreement among the two measurement techniques with a Bland–Altman plot. The Bland–Altman plot shows a small fixed bias of −0.61 s, which must be considered. The minimal detectable change (MDC) of the 5CRT differs in literature depending on the observed study population. For example, Goldberg et al. [38] found an MDC of 2.5 s in older females and Blackwood [39] a MDC of 3.54 s in older adults with early cognitive loss. Therefore, differences within the mean ± 1.96 SD might be not clinically important and the two methods may be used interchangeably.

In the examination of the durations of the single cycles and subsequent of the durations of the single transitions, we found that the first two cycles are often the fastest and the slowest cycles the last two cycles. It has to be mentioned that the 5CRT ends at the fifth standing. Therefore, the duration of cycle 5 can be influenced by the end of the test. However, since the last two cycles typically show the longest duration, this could be an indicator for a beginning physical exhaustion and could hold crucial information of changes in the functional status.

For further investigations, we should increase the repetition to confirm this result. Roldán-Jiménez et al. [40] investigated the muscular activity and fatigue of healthy adults during different repetitions (5, 10, 30) and speeds of STS tasks using surface electromyography in lower-limb and trunk muscles and found significant differences in fatigue in the *M. vastus medialis* of the quadriceps between the different STS tests, whereby an emerging EMG-activity can be reported in order of relevance for *M. vastus medialis, M. tibialis anterior, M. rectus femoris and M. erector spinae* during the sequence of seat-off [41]. The study of Roldán-Jiménez et al. affirms our presumption, whereby we assume that fatigue occurs already with small repetitions in our heterogeneous study population. In some cases, the first cycle belongs to the slowest ones. This is due to the forced starting position of the legs with an angle of 90${}^{\circ }$, which makes the rising quite exhausting. Usually, the participants change the sitting position during the test lightly to a more comfortable position for rising.

The investigation of associations between the durations of the transitions and other geriatric test results showed that, among the considered variables, only the first and average sit-to-stand duration show a significant relationship to the TUG test. This might be an indication that the evaluation of the sit-to-stand durations is a more sensitive indicator than the overall test duration or the stand-to-sit duration. The stair climb test and the walk test differ strongly from the motion sequences, whereby the TUG includes transitions and thus a relationship is more obvious than for the other tests. In contrast to our results, Goldberg et al. [38] confirmed a significant correlation between the 5CRT (overall test duration) and the TUG. We suspect that we did not find a significant correlation due to our small study population.

Additionally, we evaluated the movement patterns of the separate test cycles via a normalized cross-correlation based on the force plate data. The patterns of the same person show similar curves for the five cycles, which slightly differ in form and duration. In particular, the first and last cycle differ mostly, which seems to be an indicator of physical exhaustion as already mentioned. Another point was that the intrapersonal-variability was slightly higher for fitter participants than for slower participants. This could indicate that less fitter subjects are moving more steadily and distribute their power over the whole test duration than fitter ones, who respond well to minor deviations in the movements. The comparison of the patterns of different participants indicated the existence of different performance strategies, which is worthwhile to investigate in detail. It is known that age-related changes in the biomechanical process of standing up can be seen in adopted strategies to perform the task. For example, Gross et al. [34] revealed that elderly participants show higher muscle activity in *M. tibialis anterior* and more hip flexion prior to lift off from the chair seat in Phases 1 and 2 (see Section 3.5). We showed two examples of strategies observed in our study, which differ significantly in time and execution of movements and resulted in a higher swaying (less fit) or a higher dynamic range (fit). The comparison of performance strategies is a major topic and should be investigated in greater detail. In particular, the separate examination of the movements in the AP and ML direction could be worthwhile. In contrast to force plate measurements (measures the center of pressure), IMU measurements also allow statements about movements in anterior-posterior and mediolateral direction due to the central positioning at the hip (center of mass). Therefore, the IMU system alone can already be used as an adequate or even advantageous measurement system and the other sensors act as reference systems.

The use of other pattern matching analysis, for example dynamic time warping (DTW), could also be useful to investigate the variability in the CRT performance since this approach is considered as more robust than cross-correlation [42]. For a more detailed analysis of differences in the performance strategies, it could also be worthwhile to analyze sub-sequences in the cycle instead of the entire cycle. This would reduce the influence of the overall process and thus emphasizes differences in the sub-phases more strongly.

However, due to the limited cohort size of the current study, our results should be confirmed in a larger study population. Despite these limitations, we nevertheless believe that our findings are worthwhile for future developments in geriatric assessments and technology supported assessments, especially regarding high frequent assessments of functional decline at an early stage.

## 5. Conclusions

We introduced an approach for an automatic chair rise test detection and evaluation system via an inertial sensor integrated into a belt and a machine learning classifier. We also considered differences during the single cycles of chair rise test and therefore reveal the characteristic fatigue and unsteadiness associated with the test. Another point of this paper was the consideration of different performance strategies of older adults. We, therefore, compared the repeated movements by a single person as well as across different persons and found, from a correlations analysis, that the persons maintain their performance strategy during the test, but differ from each other inter-personally.

## Figures and Tables

**Table 1:** Scoring of the five time chair rise test regarding Guralnik et al. [19].

Score	Test Duration
4 points	≤11.19 s
3 points	11.20–13.69 s
2 points	13.70–16.69 s
1 point	16.70–60 s
0 points	>60 s or are unable to complete 5 rises

**Table 2:** Characteristics of our study population (*n* = 20) with minimum (min), maximum (max) and mean-value (mean) as well as the standard deviation (SD) of age in years, body weight in kg, body height in cm, and results for the five time chair rise test in s.

	Min	Max	Mean	SD
age [years]	72	89	78.2	4.6
weight [kg]	55.85	101.20	78.83	12.41
height [cm]	145.8	188.2	165.19	11.32
5CRT [s]	9.08	16.27	12.04	2.26

**Table 3:** Classifier configuration: method, size and step-width of the sliding window, as well as the noise reduction filter and feature set (Root Mean Square (RMS), Signal Energy (SE), Auto Correlation (AC), Correlation (C), Signal Magnitude Area (SMA), Standard Deviation (SD)). The used data for each feature are specified in brackets at the end of the line: Acceleration data (Acc), Gyroscope data (Gyro). The abbreviations HL and HN stand for hidden layer and hidden nodes. The cut-off frequency of the specific filters is $f_{c}$.

Classifier	Method	Window Size (s)	Step Width (s)	Filter	Feature-Set
State	Boosted Decision Trees	1.405	0.072	Low pass ($f_{c}$ = 6.1 Hz)	AC, C, Mean (Acc), RMS, SD, SE (Acc + Gyro)
Static	Multilayer Perceptrons (5 HL, 7 HN)	2.511	0.427	-	Mean, SMA (Acc), Pitch, AC, C (Acc + Gyro)
Transition	Multilayer Perceptrons (4 HL, 40 HN)	1.135	0.073	Low pass ($f_{c}$ = 4.5 Hz)	RMS (Acc), Mean, SE (Gyro), AC, C, SMA, SD (Acc+Gyro), Pitch

**Table 4:** *P*-values of our linear regression analysis of the chair rise variables and the Timed Up and Go test (TUG), the walk test of the Frailty Criteria and the Stair Climb Power Test (SCPT). Significant relationships are marked with an asterisk.

Duration of	TUG	Walk Test	SCPT
1. sit-to-stand	0.02 *	0.09	0.75
1. stand-to-sit	0.39	0.26	0.23
last sit-to-stand	0.22	0.46	0.88
last stand-to-sit	0.17	0.11	0.32
average sit-to-stand	0.02 *	0.09	0.89
average stand-to-sit	0.22	0.32	0.64
five time chair rise test	0.59	0.67	0.71

**Table 5:** Descriptive statistics (minimum (min), maximum (max), standard deviation (SD)) of correlation coefficients of the first test cycle with the following cycles.

Cycle	1–2	1–3	1–4	1–5
min	0.75	0.8	0.79	0.71
max	0.98	0.99	0.99	0.99
mean	0.95	0.94	0.93	0.92
median	0.96	0.96	0.96	0.96
SD	0.06	0.05	0.07	0.08
range	0.23	0.19	0.20	0.28

**Table 6:** Descriptive statistics (minimum (min), maximum (max), standard deviation (SD)) of correlation coefficients of the first test cycle of different participants.

Min	Max	Mean	Median	SD	Range
0.65	1	0.88	0.88	0.09	0.34
